# Effect of guided implant placement learning experiences on freehand skills: A pilot study

**DOI:** 10.1002/cre2.878

**Published:** 2024-03-20

**Authors:** Caroline Carrico, Lenart Skrjanc, Domen Kanduti, George Deeb, Janina Golob Deeb

**Affiliations:** ^1^ Dental Public Health and Policy, School of Dentistry Virginia Commonwealth University Richmond Virginia USA; ^2^ Dentalni Center Celesnik Bled Slovenia; ^3^ Department for Oral Diseases and Periodontology, Division for Dental Medicine, Faculty of Medicine University of Ljubljana Ljubljana Slovenia; ^4^ Department of Oral and Maxillofacial Surgery, School of Dentistry Virginia Commonwealth University Richmond Virginia USA; ^5^ Department of Periodontics, School of Dentistry Virginia Commonwealth University Richmond Virginia USA

**Keywords:** accuracy, freehand, guided navigation, implant

## Abstract

**Objectives:**

Guided implant systems can be used as a training approach for placing implants. This in vitro prospective randomized pilot study evaluated the learning progression and skill development in freehand placement of two implants supporting a three‐unit fixed prosthesis on a simulation model among novice operators.

**Material and Methods:**

Four senior dental students with no prior implant placement experience participated in the study. As a baseline, each student placed two mandibular and two maxillary implants by freehand technique on a simulation model. Sixteen consecutive guided placements using a static guide, dynamic navigation, and template‐based guide followed totaling 32 guided implant placements into maxillary and mandibular models. Freehand implant placements before and after the various guided navigation attempts were compared to assess their impact on freehand skill. Metrics compared included surgical time, horizontal, vertical, and angulation discrepancies between the planned and placed implant positions measured on superimposed CBCT scans and analyzed with repeated measures regression with Tukey's adjusted pairwise comparisons (*α* = .05).

**Results:**

Before training with guided techniques, the average baseline freehand implant placement took 10.2 min and decreased to 8.2 after training but this difference was not statistically significant (*p* = .1670) There was marginal evidence of a significant difference in the 3D apex deviation with an average improvement of 0.89 mm (95% CI: −0.38, 2.16, *p* = .1120); and marginal evidence of a significant improvement in the overall angle with an average improvement of 3.74° (95% CI: −1.00, 8.48, *p* = .0869) between baseline and final freehand placement attempts.

**Conclusions:**

Within the limitations of this pilot study, guided implant placement experiences did not significantly benefit or hinder freehand placement skills. Dental students should be exposed to various placement techniques to prepare them for clinical practice and allow them to make informed decisions on the best technique based on their skills and a given clinical scenario.

## 1 INTRODUCTION

The aim of successful implant treatment is to achieve functional and aesthetic rehabilitation of the missing dentition. Osseointegration and an optimal implant position in relation to the adjacent teeth and anatomical structures are necessary for a predictable result (Kopp et al., 2012). Implant malpositioning can be related to a variety of complications such as bone loss and soft tissue dehiscences, damage to adjacent anatomical structures, and compromised restorative outcomes (Kopp et al., 2012; Romanos et al., 2019). Placing implants accurately into the planned position and visualizing how the implant position will affect the final prosthesis can be challenging for an operator with limited experience. Using guided technique based on a preoperative three‐dimensional (3D) virtual plan of prosthesis and implants can minimize the possibility of large deviations during implant placement. Use of guided surgical techniques may also improve the haptic feedback, perception for spatial distribution, and can aid in development of hand–eye coordination necessary for drill positioning and orientation. This is particularly critical for larger prostheses supported by multiple implants that require accurate and correct implant spacing and angulation (Block et al., 2017; Deeb et al., 2017; Scherer et al., 2014; Tahmaseb et al., 2018). Unexpected intraoperative complications, such as guide fracture or navigation failure, and limitations including mouth opening, costs, available equipment can render guided surgery not possible. Under such circumstances, the operator should be able to rely on their freehand abilities and visualization. While the accuracy of the freehand implant placement may be sufficient for most clinical situations (Brief et al., 2005) it can present a challenge for an operator without experience (Choi et al., 2017; Payer et al., 2008).

Implant placement accuracy is measured as entry distance deviations, apical distance deviations, and angular deviation between planned and placed implant position (Block & Emery, 2016; Block et al., 2017; Cassetta & Bellardini, 2017; Somogyi‐Ganss et al., 2015; Van Assche et al., 2012). The placement of individual implants with dynamic navigation was deemed feasible for novice operators. Dental students with no previous implant placement experience can learn to perform single implant placement competently and accurately with guidance of dynamic navigation after only three attempts (Golob Deeb et al., 2019). When using static CAD/CAM fabricated guides, predictable results can be obtained from the first attempt rather than identifying a typical learning curve (Cassetta & Bellardini, 2017). Despite the data showing that practitioner's experience in placing implants is an important factor when evaluating accuracy for freehand placement (Choi et al., 2017), there is limited evidence evaluating the amount of training needed for the predictable result.

The accuracy of various guiding techniques has been previously studied. Guided implant placement techniques were found to be significantly more accurate in comparison to freehand technique and computer‐guided dynamic navigation have been found comparable to that of static guidance (Block et al., 2017; Deeb et al., 2022). Both static and dynamic guided methods were found significantly more accurate than a conventional template‐based guide (TBG) technique (Deeb et al., 2022). While experienced operators rely on their expertise when using less accurate placement methods, novice practitioners entering the field of implantology do not have that advantage.

Cases requiring multiple implants for a fixed partial denture (FPD) are even more challenging as deviations are compounded by the dependency of the implants' positions on each other. Implants have to be placed at the right angles and optimally spaced for an optimal restorative outcome. Guided techniques may provide an inexperienced operator with additional support as the skills and confidence are acquired in addition to exposure to new and varying techniques.

This prospective randomized pilot study aimed to evaluate the impact of experiences with guided implant placement for a two‐implant supported three‐unit FPD on a simulation model among inexperienced dental students. Initial freehand implant placement attempts before any prior implant placement experience were compared to the last freehand placement attempt that followed training with guided methods on maxillary and mandibular simulation models. The accuracy and efficacy of the initial and final attempts would be compared to determine if the guided navigation experiences had an impact on freehand skill either through improvements or potential dependencies for the student. We hypothesized that the guided navigation experiences would improve freehand skills through repeated proprioceptive feedback.

## 2 MATERIALS AND METHODS

This was an in vitro pilot study which was reviewed and approved by the Institutional Review Board at Virginia Commonwealth University (HM20011878). Four final‐year dental students (two female and two male) with no previous experience in dental implant surgery (novice operators) participated in the study after informed consent.

**Figure 1:**
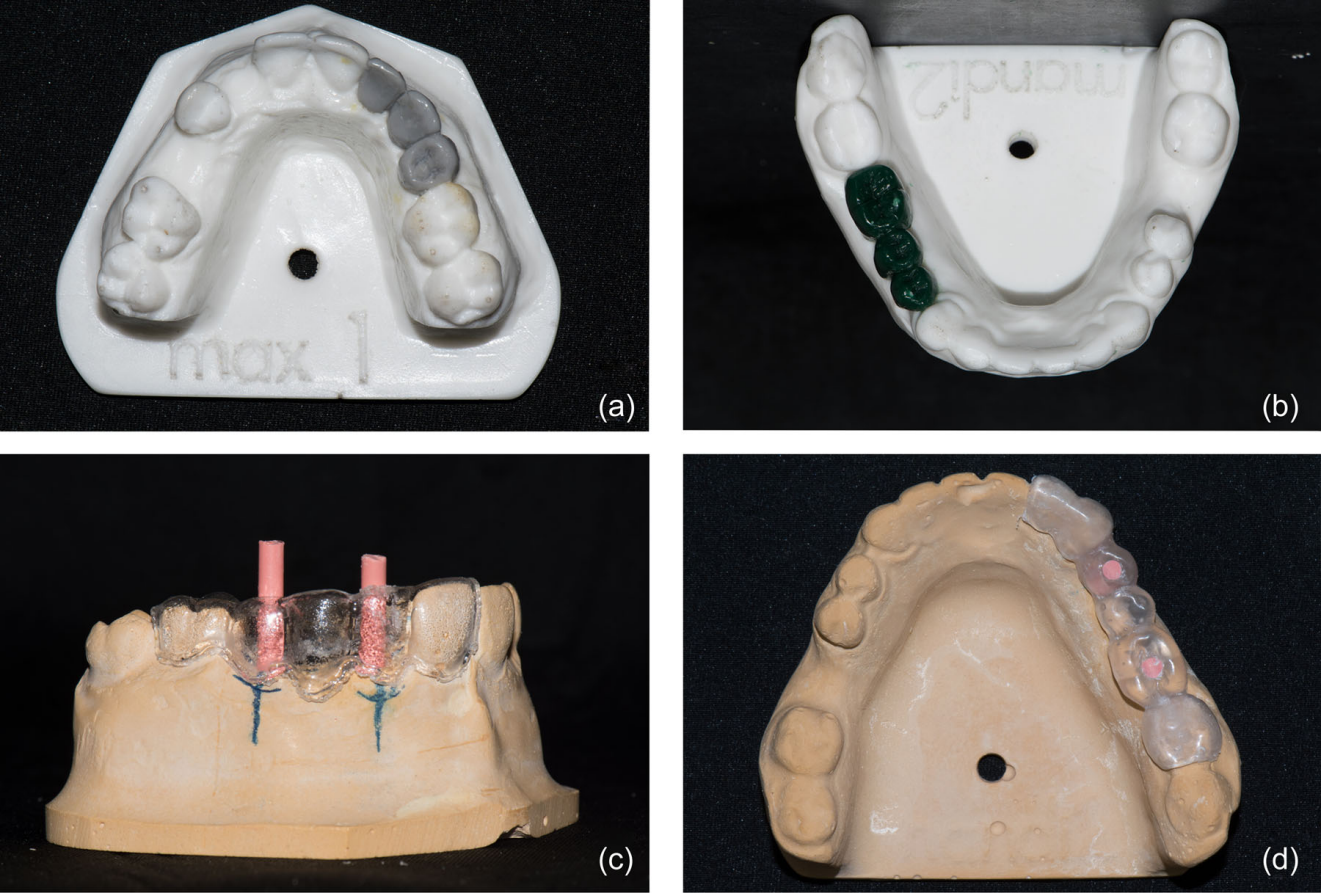
(a) maxillary PMMA model with wax up; (b) mandibular PMMA model with wax up; (c) maxillary model during radiographic guide/stent production—gutta‐percha insertion for planning implant position/axis (d) final radiographic guide/stent on mandibular model.

Simulation polymethylmethacrylate (PMMA) 3D printed maxillary and mandibular models were used. Two implants for a three‐unit FPD were placed in each attempt. The maxillary model was missing anterior teeth on the left side (second incisor, canine, first premolar), while mandibular model was missing posterior teeth on the right side (first and second premolar, first molar). A diagnostic wax‐up was performed on both PMMA models simulating the final prostheses to guide accurate implant planning. Models with wax‐up were duplicated in type four stone (Suprastone, Kerr) (Figure 1). A pressure mold was made from 2.0 × 125 mm thermoplastic foil (Scheu—Dental) for radiographic and TBG guide production (Figure 1). The optimal position of the access hole of the planned implant crown was determined. Implant position was planned and marked on the duplicated jaw model. According to the marked position, access hole and the axis of the adjacent tooth on an optimal implant insertion line was determined using laboratory surveyor. Insertion lines for both implants supporting the FPD were paralleled and two gutta‐percha bars (Figure 1) were secured inside the thermoplastic template with acrylic resin (Biocryl ICE). Optimal implant position was determined by an experienced board certified Periodontist (JGD) and Oral surgeon (GD). Each model was tagged with four fiducial markers placed on the buccal aspect apically to the central incisors and on the posterior aspect of the model to ensure the 3D orientation of the model in the CBCT and enable accurate superimposition. A preoperative CBCT scan (iCAT FLX V10, Imaging Sciences International LLC, Kavo) of the model was taken with a radiographic guide and fiducial markers in place. The data from CBCT were loaded into the Navident (ClaroNav, Ontario, 116 Canada) dynamic guidance system software where virtual implant planning was performed.

All implants were placed based on the manufacturer's recommendations and proposed osteotomy protocol. At missing premolar and incisor sites, tapered implants with 13 mm length and 3.7 mm diameter and at molar sites, tapered implants with 13 mm length and 4.7 mm diameter were used (Tapered Screw‐Vent MTX, Zimmer Biomet). The initial osteotomy was made using a pointed starter drill (850 rpm, Torque 25 Ncm) and was followed by manufacturers proposed drill sequence for dens For accuracy measurements, a computer software Evalubone (600–800 rpm, 25 Ncm). Before implant insertion, the osteotomy site was threaded using a cortical bone tap in conjunction with ratchet. Implant was gently seated into the osteotomy and was inserted using a handpiece with 20 rpm and torque set to 35 Ncm. Final depth of implant and proper orientation was obtained using Ratchet with Torque not exceeding 35 Ncm.

**Figure 2:**
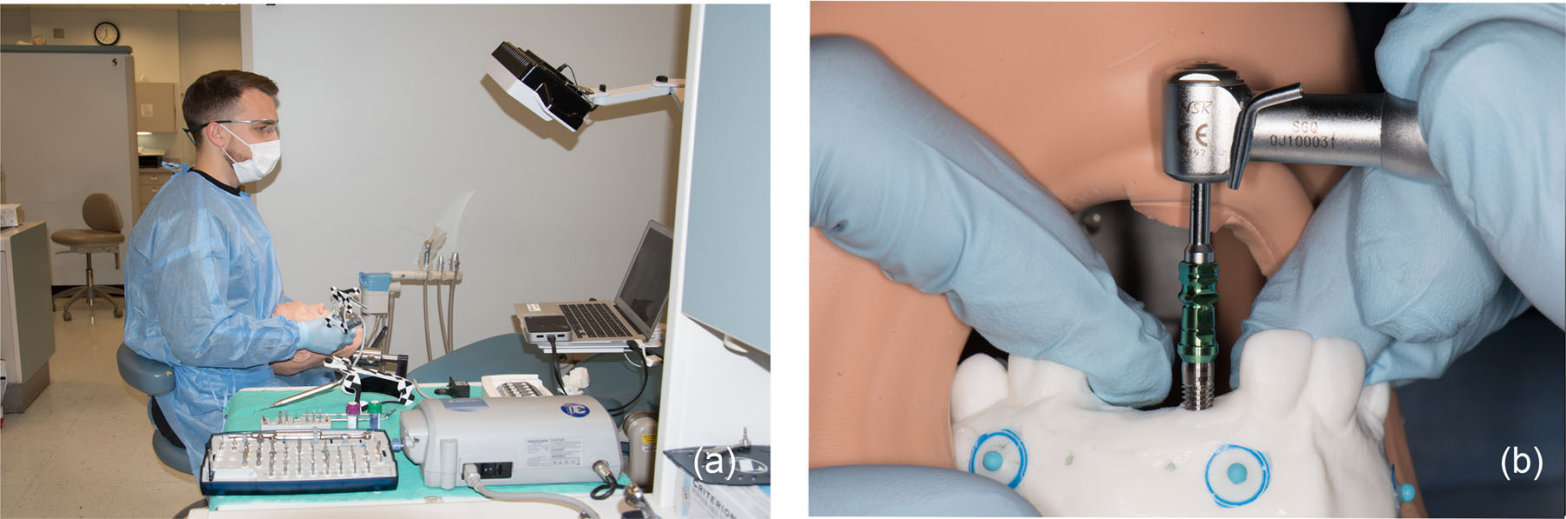
(a) Manikin head and implant placement experimental set‐up simulating clinical situation, (b) implant insertion on model secured in manikin head.

**Figure 3:**
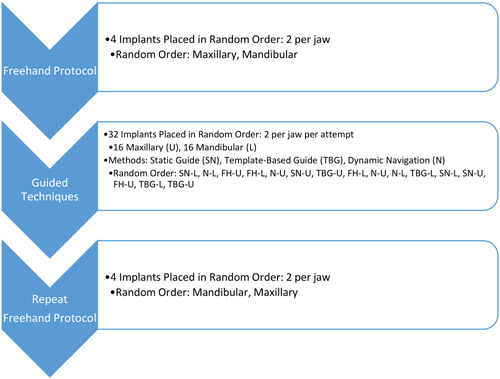
Flowchart of implant placement techniques.

Before implant placement, operators were introduced to the study protocol, placement methods, and equipment. Operators aimed to place the implants in the closest accordance with planned positions using four different techniques. All operators were instructed to not use excessive pressure during osteotomy and use a passive drilling method was recommended for all dental procedures. Jaw models were marked according to the guidance method secured in the manikin head on a dental chair to simulate clinical situations. Each operator could adapt dental chair and manikin head to optimal ergonomic posture for good accessibility, optimal visual field, and neutral posture (Figure 2). All operators were sitting during implant placements using 8–12 o'clock working positions. Each operator started the study by placing two implants in a maxillary and mandibular model following freehand protocol, which was used as a baseline. Placements using guided techniques followed a computer‐generated randomized schedule for each implant site and placement attempt. During this portion of the study, two additional freehand attempts were included per jaw and were randomized within the guided attempts. Guided techniques included a static guide, dynamic navigation, and a template‐based guide. Each operator placed 32 implants, 16 in each jaw. After completing the guided navigation attempts, each operator repeated the initial freehand placement of two implants in both the maxillary and mandibular models. In total, each operator placed 40 implants into 10 maxillary and 10 mandibular models. A study flowchart is provided in Figure 3. For comparison of baseline and final freehand implant placement, the final sample size for four operators included 32 attempts, 16 before (baseline freehand attempt) and 16 after (final freehand attempt) evenly distributed between maxillary and mandibular models. Surgical time, horizontal, vertical, and angulation discrepancies between the planned and placed implant positions were assessed and analyzed with repeated measures regression with Tukey's adjusted pairwise comparisons (*α* = .05). This design was estimated to have 80% power to detect an effect size of at least 0.8 with a 5% two‐sided significance level. This effect size would indicate clinically and statistically significant changes in the efficacy and accuracy and determine if a larger study would be warranted.

### 2.1 Implant placement protocols

**Figure 4:**
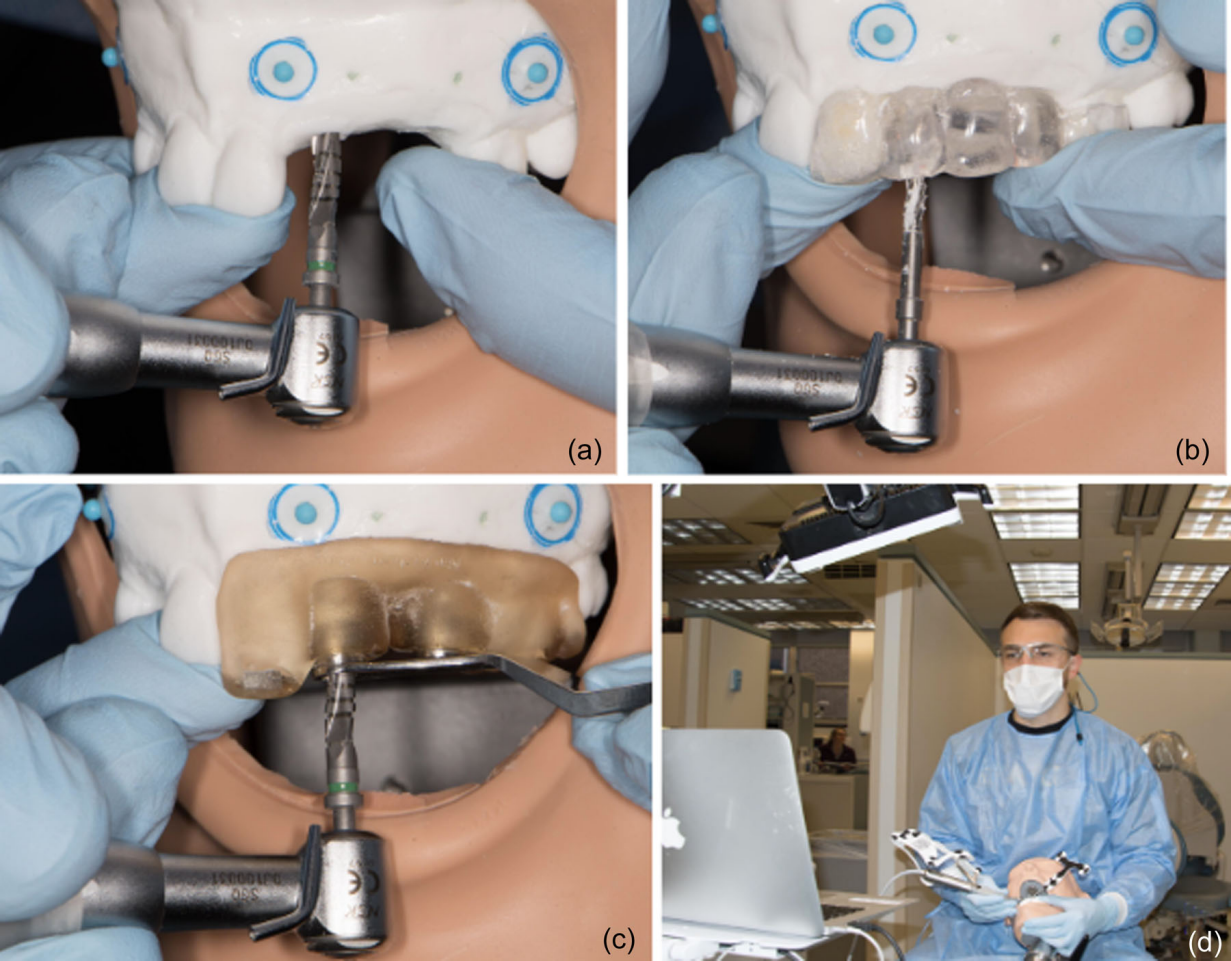
(a) Freehand implant placement; (b) implant placement using TBG; (c) implant placement using static guide; (d) implant placement when using dynamic navigation.

- Freehand protocol (FH): No surgical guidance was used. Students planned and placed implants according to the virtual CBCT plan using radiographic guides and laboratory wax‐up for visual orientation. Throughout the FH attempts, virtual plans on CBCT were available for visual reference and guidance. Any needed measurements were taken on computer screen (Figure 4a).
- Template‐based guide (TBG): The acrylic radiographic guide based on wax‐up of the proposed restoration was converted into a surgical guide by removing Gutta‐percha and enlarging guide holes to 3 mm in diameter. Opening access to the buccal surface of the mandibular and the palatal surface of the maxillary guides were made. Operators used TBG for narrow drill (pilot drill) osteotomy. The enlargement of the osteotomy and implant placement was performed without the guide (Figure 4b).
- Static guide (SG): 3D scan of the model was made using intraoral scanner (Trios, 3Shape). CBCT data and intraoral scan were imported into CAD software (Implant Studio, 3Shape). Virtual implants were selected and positioned based on restorative superstructures and available space. The surgical guide was designed, processed according to manufacturer's guidelines, and 3D‐printed using surgical guide resin (Form 2, Formlabs). Guides were washed in 99% Isopropyl alcohol for 10 min (Form 140 Wash, Formlabs), dried and post cured for 30 min at 60°C (Form Cure, Formlabs). Metallic sleeves (Zimmer, Biomet) were inserted into guide holes to enable rigid drill stabilization during implant placement. For implant placement, a guided surgical kit and guiding tubes (Zimmer Biomet) were used according to the manufacturer's guidelines throughout the implantation process (Figure 4c).
- Dynamic navigation (DN): CBCT data of models tagged with fiducial markers and radiographic guide was imported into navigation system software (Navident, Claronav). Virtual implants were selected and positioned based on restorative superstructures and available space. Both model and the patterned jaw tag were tightly secured onto the mannequin. The Navident device was positioned in front of the operator, and the optical camera was secured above the operating field. A tracking stereoscopic camera tracks a spatial relationship between the jaw‐tag secured on a model, drill‐tag on the handpiece, and the tag on the tracing tool. Before starting the osteotomy preparation, calibration for each drill was performed on the calibration tag. Appropriate positioning of the mannequin, camera, and computer‐enabled easy visualization of the computer screen displaying real‐time feedback of the actual drill position during osteotomy preparation in relation to the planned implant position (Figure 4d).

## 3 MEASUREMENTS AND ANALYSIS

Freehand implant placements before and after the various guided navigation attempts were compared to assess the impact of guided navigation on freehand skills. Efficacy was defined and evaluated as time spent on the actual implant placement. Time was measured for every attempt by a third party using a stopwatch. Time began with a “Start” announcement by the timer and ended when the procedure was completed and instrument was placed on the table. The average time per implant and per model (maxillary or mandibular) was calculated and compared among initial and final attempts.

**Figure 5:**
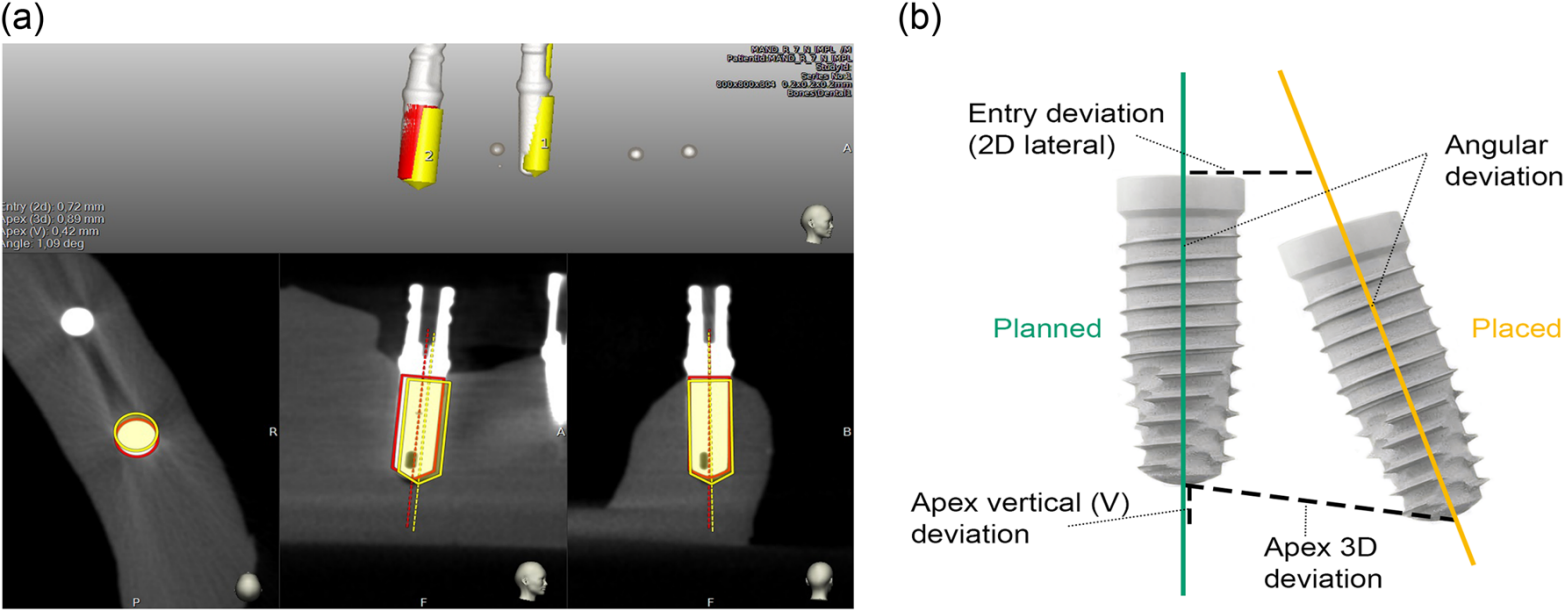
(a) Software (EvaluNav) superimposition of pre‐ and postoperative CBCT; (b) measured deviations.

Accuracy was defined and assessed by comparing the planned and placed implant positions and calculating deviations at the (Figure 5b):

- entry deviation (lateral 2D)—lateral deviation of the cervix
- apical vertical deviation (V)—height deviation of apex—difference between apex of planned and placed implant
- apical 3D deviation—deviation between apex of planned and placed implant taking into account entry, apical vertical and angular
- angle deviation—deviation of the long axis of placed implant according to planned position.

For accuracy measurements, a computer software EvaluNav (Navident, Claronav) was used (Figure 5a). Software allows for the comparison in position of planned and placed implants. Pre and postoperative CBCT scans of models were taken using the same settings (voxel size of 0.2 mm, 7.4 s exposure time, 120 kVp, 5 mA and 611.6 mGy/cm 2, FOV: 8 ×6 cm). A preoperative CBCT scan of the model with virtually planned implants and a postoperative CBCT scan with placed implants were imported to the EvaluNav superimposition program. Since PMMA models have low radiographic opacity which makes superimposition for surface matching based on anatomical features more difficult and less reliable, fiducial makers were placed on the model bilaterally to ensure precision of the superimposition process (Figure 2) (Choi et al., 2020). Four fiducial markers on each model were used as matching points during superimposition between the two CBCTs to orient and match the pre and post‐op scans and superimpose them through a best fit algorithm (Lv et al., 2023; Yang et al., 2015). Trueness is assured through accuracy of superimposition by aligning the fiducial markers on the models. Precision is assured as the process is not dependent on provider judgment rather through an objective, software generated analysis/algorithm. After the superimposition, the software calculates the entry deviation, apical vertical deviation, apical 3D deviation and angle deviation between planned and placed implant positions based on the aforementioned superimposition. Average deviations were later calculated for each implant and compared among initial and final freehand implant placement attempts.

### 3.1 Statistical methods

Changes in procedure time and deviations at the implant level were compared using repeated measures analysis of variance to account for the four different operators. Post hoc pairwise comparisons were adjusted using Tukey's adjustment. Significance level was set at 0.05 and SAS EG v.8.2 (SAS Institute) was used for all statistical analyses and to generate randomization schedule.

## 4 RESULTS

At the initial freehand attempt, the average implant placement took 10.2 min, and decreased to 8.2 after training, but this difference was not statistically significant (*p* = .1670, Table 1). There was marginal evidence of a significant difference in the 3D apex deviation, which had an average improvement of 0.89 mm (95% CI: −0.38, 2.16, *p* = .1120). There was also marginal evidence of a significant improvement in the overall angle, which improved by an average of 3.74° (95% CI: −1.00, 8.48, *p* = .0869). The differences in the 2D entry (*p* = .9839) and vertical apex (0.7057) deviations were minimal and not statistically significant (Table 1).

**Table 1:** Mean (SE) drilling time and deviations before and after guided navigation training exercise.

	Before	After	Difference	*p* Value
Drill time (min)	10.2, 0.78	8.2, 0.78	2.00, 1.10	.1670
2D entry (mm)	1.1, 0.18	1.1, 0.18	0.01, 0.26	.9839
3D apex (mm)	2.7, 0.28	1.8, 0.28	0.89, 0.40	.1120
Vertical apex (mm^3^)	0.3, 0.06	0.3, 0.06	−0.04, 0.09	.7057
Angle (°)	9.5, 1.05	5.7, 1.05	3.74, 1.50	.0869

**Figure 6:**
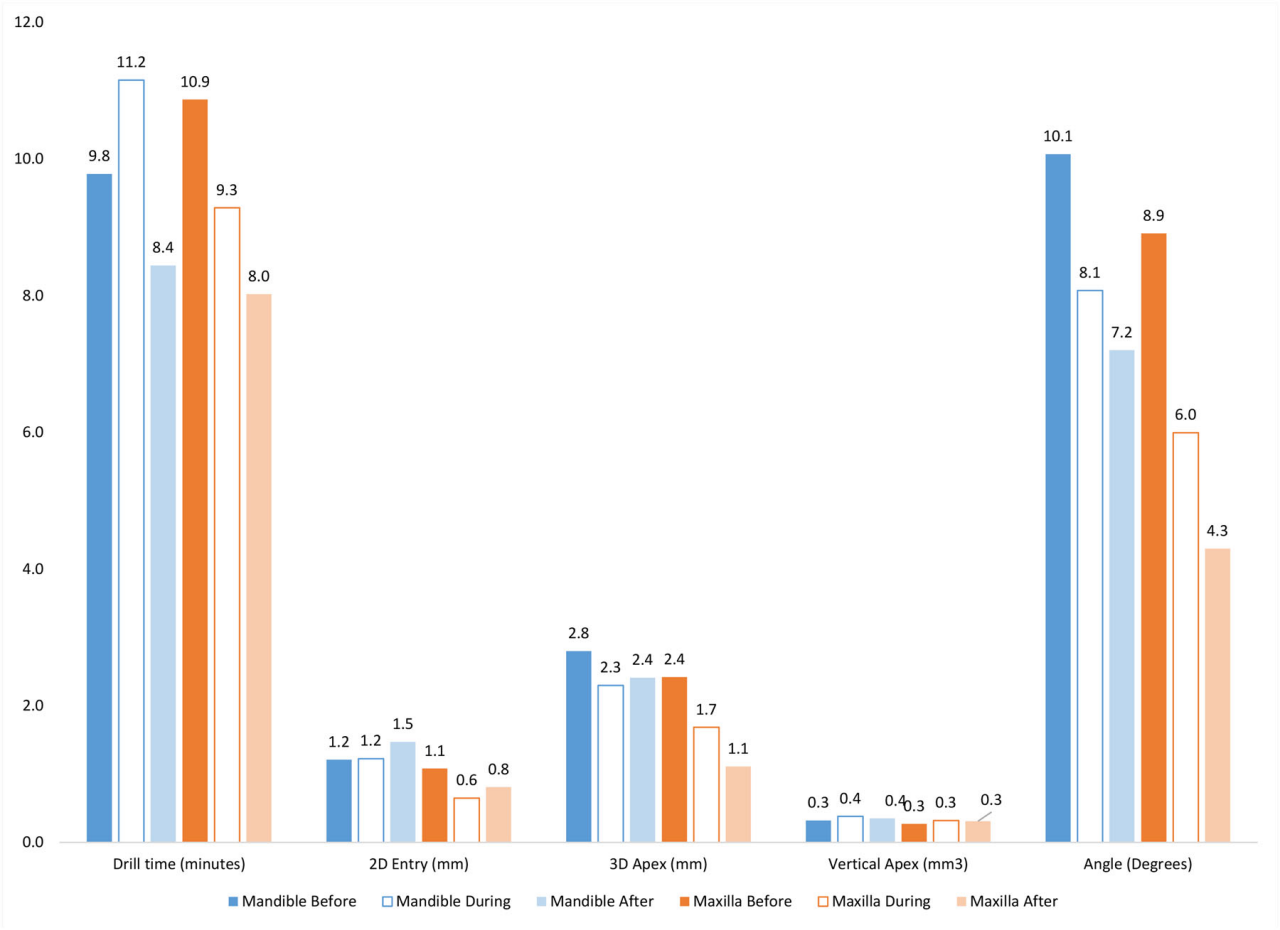
Mean (SE) freehand drilling time and deviations before, during, and after guided training exercise.

These results were similar when analyzed by jaw (Table 2). Of particular note is the change in Apex 3D which was on average 0.47 mm (95% CI: −1.25 to 2.19) for implants on the mandible but 1.31 mm (95% CI: −0.29, 2.91) for the maxilla. Neither difference was statistically significant (*p* = .4519, .0794, respectively) nor was the difference in improvement between the two jaws (*p* = .3350). Figure 6 displays the average time and deviations by jaw) before and after the guided navigation attempts. The average value for the freehand attempts during the guided navigation portion of the study is also displayed in the figure to demonstrate consistency in freehand placements.

**Table 2:** Mean drilling time and deviations before and after guided navigation training exercise by jaw (mean, SE).

	Mandible			Maxilla		
	Before	After	*p* Value	Before	After	*p* Value
Drill time (min)	9.78, 0.91	8.44, 0.91	.3574	10.87, 1.31	8.02, 1.31	.2484
2D entry (mm)	1.21, 0.30	1.47, 0.30	.5809	1.08, 0.19	0.81, 0.19	.3746
3D apex (mm)	2.80, 0.38	2.41, 0.38	.4519	2.42, 0.35	1.11, 0.35	.0794
Vertical apex (mm^3^)	0.32, 0.10	0.35, 0.10	.8447	0.27, 0.08	0.31, 0.08	.7284
Angle (°)	10.07, 1.49	7.20, 1.49	.2667	8.91, 1.48	4.30, 1.8	.1146

## 5 DISCUSSION

This pilot study evaluated the learning progression and skill development by comparing a freehand implant placement before any other implant placement experience and compared it to placement following training with different guided methods on a simulation model.

Implant placement accuracy of freehand versus guided placement methods performed by experienced clinicians has been studied previously. In general, the static fully guided technique provides the most accurate results in comparison to half guided and freehand approaches (Gargallo‐Albiol et al., 2020). A systematic review reported that statically guided implant placement resulted in an average entry deviation of 1.2 mm, average 3D apical deviation of 1.4 mm, and an average angular deviation of 3.5° (Tahmaseb et al., 2018). Implants placed via dynamic navigation reported averages for entry deviation of 0.81 mm, apical deviation of 0.91 mm, and a range of angular deviations from 0.90° to 3.81° (Pellegrino et al., 2021). According to its accuracy, dynamic navigation can be used for most clinical implant situations (Afrashtehfar et al., 2022). Studies evaluating freehand implant placement accuracy found similar average deviations. Varga et. al. reported entry deviation of 1.82 mm, average 3D apical deviation of 2.43 mm, and average angular deviation of 7.03° (Varga et al., 2020). Block et al. reported average entry deviation of 1.15 mm, apical depth deviation of 0.92 mm, 3D apical deviation of 2.51 mm, and angular deviation of 7.69 for freehand (Block et al., 2017). Despite the fact that this study was done with novice operators, the results are consistent with the previously published observations (Afrashtehfar et al., 2022; Gargallo‐Albiol et al., 2020; Hama & Mahmood, 2023; Pellegrino et al., 2021; Schnutenhaus et al., 2021; Varga et al., 2020). Interestingly, the deviations were consistent with other studies where trained surgeons performed implant placements.

Even though literature suggests that guided implant surgery should be the preferred implant placement method to increase accuracy, using a guide is not always feasible, especially in cases with limited access and mouth opening or when guide fails (Block & Emery, 2016). Another disadvantage of using a static guide is the inability to adapt during the surgery which can affect implant position if surgical site doesn't resemble situation on presurgical CBCT (Block & Emery, 2016). On the other hand, freehand implant placement can lead to clinically acceptable results when appropriate presurgical planning, 3D imaging, and case selection are exercised (Choi et al., 2017; Schnutenhaus et al., 2021). And further, additional studies have demonstrated little to no differences in patient‐reported outcomes based on the placement technique (Afrashtehfar, 2021; Engkawong et al., 2021).

Many factors can influence implant final position after guided or freehand surgery including operator experience, drilling method and speed, bur diameters, surgical guide design, implant location, number and proximity of adjacent teeth, number of placed implants, and anatomical factors. This study was conducted in a controlled and standardized dental manikin condition controlling for most influencing factors. All operators were novices without previous experience in implant placement. We used a manikin head mounted on a dental chair to closely simulate clinical conditions. All operators were right‐handed, and they all placed implants in models with the same edentulous sites to limit potential. Despite controlled environment, drilling pressure, operator posture, working position, and operator 3D visualization abilities could have played a significant role in implant placement accuracy. These individual‐specific factors were not evaluated or controlled for in this study and could be further examined in future research.

Fine motor skills and spatial assessment acuity are necessary skills for placing implants accurately into optimal positions. These motor skills are obtained through training and practice and may be enhanced by using guided methods that subconsciously help develop haptic feedback and a sense for spatial orientation. Any planned movement consists of the formation of a movement goal, linking that goal to the appropriate action, and precise execution and maintenance of that selected action despite perturbations being present. Improvement at any step of that pathway is formally considered motor learning (Krakauer et al., 2019). The most common motor‐learning model described in the literature relies on the observation of others, especially skilled individuals, before performing a procedure independently (Harris et al., 2018; Shea et al., 2000) and on gradual movement adjustment based on outcome feedback (Wolpert et al., 2011; Wulf, 2013). Approaches to improving motor learning in medicine have changed considerably over the years due to advances in technology including the use of computer‐based video instructions (Xeroulis et al., 2007), virtual reality simulation (Seymour, 2008), and dynamic navigation (Casap et al., 2011).

Repetition of a motor task results in storage and consolidation of information in long‐term memory creating new neural pathways in the central nervous system (Čoh et al., 2004). This ongoing process called neuroplasticity enables the central nervous system to adapt and modify to changing environments and demands (Neville & Bavelier, 2002). Body movement that is repeated enough times and is supported by visual, somatosensory, and proprioceptive feedback may lead to plastic changes—reorganization of the motor cortex so that future movements can be executed more precisely and effortlessly (Pascual‐Leone et al., 1994). Learning is directly associated with the context of the learning skill, a process known as the specificity of the learning principle. The specificity of learning a principle is strongly connected to the learning context of the developing skill that an individual learns during training (Bachman, 1961).

Meanwhile, recent data, that provide background for this pilot, challenge the specificity of learning. It has been reported that subjects are able to transfer the adaptation across similar tasks, thus facilitating and accelerating motor learning (Roller et al., 2001). Furthermore, transferability occurs not only between resembling tasks but also among diverse motor tasks, when individuals are engaged in multiple learning experiences (Seidler, 2004).

In this pilot study, the transferability and adaptation across similar tasks may have contributed to improving motor skills in spatially challenging freehand placement of two nonadjacent implants that need to be parallel and at an ideal distance to accommodate a fixed prosthesis. While the four techniques involved different approaches to placing implants, the tasks were similar and skills transferred across tasks. Freehand implant placement without any guidance was likely not significantly, negatively impacted due to neuroplasticity and proprioceptive spatial feedback acquired despite the presence of the guided tools. Accuracy and efficiency between the first and last freehand implant placement attempt likely improved through the optimization learned through using dynamic and static guidance.

While differences in the 2D entry and vertical apex deviations demonstrated no clinically meaningful changes, these measures are inherently easier for a novice operator to visualize and align with the treatment plan. However, the overall angle and 3D apex deviations are more difficult to visualize as they account for deviations in all dimensions and compound along the implantation path. These measures demonstrated potentially, clinically meaningful differences with the data at hand, with differences of nearly 1 mm at the apex and 4°. Especially in the clinical application of a multiunit prosthesis where deviations compound due to the interdependencies of the individual units. With the limited sample size in this study, there was approximately 60% power to detect the observed differences in 3D apex (62%) and overall angle (65%) deviations. Assuming continued results, the differences would be statistically significant with an additional two students (i.e., 24 total implants placed).

Different digital methods are available for assessing implant position after placement. Digital registration using postsurgical optical scanning provides similar results in evaluating implant position accuracy as conventional CBCT (Yi et al., 2022). In this study, the evaluation of implant placement accuracy was done using pre‐ and postsurgical CBCTs and as an in vitro study not subject to a radiation exposure limitation. The same settings were used for all models and were adjusted for optimal resolution of images. The PMMA models are less radiopaque and using anatomical marks could introduce a less reliable alignment thus fiducial markers were used to ensure precise alignment and superimposition of pre‐ and postsurgical CBCT. According to selected settings, voxel resolution did not allow for superimposition precision better than 0.2 mm. In this study, an automated software superimposition and deviation analysis tool EvaluNav (Navident, Claronav) was used to provide standardized analysis. Superimposition and measurement parameters were preset by the software company and could not be changed during the evaluation process which eliminated bias during analysis.

The in vitro design of this study might limit the generalization of the results to clinical situation. Clinical environment is different in terms of soft tissues, bleeding, visibility, different bone density, mouth opening, patient movements, and patient adoption to procedure. PMMA model structure differs from natural bone and gives operator different tactile feedback. Debris produced from the drilling can accumulate in the implant osteotomy site and can lead to discrepancies in the implant final position. Another limitation of this study is the small sample size which did not allow for statistical evaluation and comparison of each single implant site separately. From the observation of the result, we noticed that the most challenging implant osteotomy was lower right premolar because of the alveolar ridge curve toward molar region. The entry point was on a steep part of the ridge which led to possible displacement of pilot drill during initial osteotomy. Additionally, all operators were right‐handed so the results may not apply to left‐handed operators. This pilot also lacked a freehand‐only group which may have demonstrated a different learning curve. Further research evaluating different variables and possible learning curve of freehand implant placement is needed.

Freehand implant placement accuracy maintained and slightly improved through repeated attempts as learning through different guiding techniques likely provided adaptation across tasks. Optimization of drill and implant position may have been achieved through guided experiences that facilitated the execution of preplanned implant placement in an optimal position. Through repeated guided attempts for placement of the two implants supporting a three‐unit FPD, motor, and perception skills evolved to enable an increase in efficiency and accuracy without developing dependencies.

## 6 CONCLUSION

Within the limitations of this pilot study, guided implant placement experiences did not significantly benefit or hinder freehand placement skills. Dental students should be exposed to various placement techniques to prepare them for clinical practice and allow them to make informed decisions on the best technique based on their skills and a given clinical scenario.

## AUTHOR CONTRIBUTIONS

*Study design*: Caroline Carrico, Lenart Skrjanc, Domen Kanduti, George Deeb, and Janina Golob Deeb. *Data management*: Caroline Carrico. *Data analysis and interpretation*: Caroline Carrico. *Writing of the main manuscript*: Caroline Carrico. *Data acquisition*: Lenart Skrjanc and Domen Kanduti. *Interpretation of results*: Lenart Skrjanc, Domen Kanduti, and Janina Golob Deeb. *Writing and revising manuscript*: Lenart Skrjanc, Domen Kanduti, and Janina Golob Deeb. *Materials allocation*: George Deeb. *Critical review of results and manuscript*: George Deeb. *Study oversight*: Janina Golob Deeb. *Study concept*: Janina Golob Deeb.

## CONFLICT OF INTEREST STATEMENT

The authors declare no conflict of interest.

## Data Availability

Research data are not shared due to Institutional Review Board approval restrictions.

## References

[cre2878-bib-0001] Afrashtehfar, K. I. (2021). Conventional free‐hand, dynamic navigation and static guided implant surgery produce similar short‐term patient‐reported outcome measures and experiences. Evidence‐Based Dentistry, 22(4), 143–145. 10.1038/s41432-021-0216-9 34916642

[cre2878-bib-0002] Afrashtehfar, K. I. , Jurado, C. A. , & Moshaverinia, A. (2022). Dynamic navigation may be used for most implant surgery scenarios due to its satisfactory accuracy. Journal of Evidence‐Based Dental Practice, 22(4), 101797. 10.1016/j.jebdp.2022.101797 36494104

[cre2878-bib-0003] Van Assche, N. , Vercruyssen, M. , Coucke, W. , Teughels, W. , Jacobs, R. , & Quirynen, M. (2012). Accuracy of computer‐aided implant placement. Clinical Oral Implants Research, 23(Suppl 6), 112–123. 10.1111/J.1600-0501.2012.02552.X 23062136

[cre2878-bib-0004] Bachman, J. C. (1961). Specificity vs. generality in learning and performing two large muscle motor tasks. Research Quarterly. American Association for Health, Physical Education and Recreation, 32(1), 3–11. 10.1080/10671188.1961.10762064

[cre2878-bib-0005] Block, M. S. , & Emery, R. W. (2016). Static or dynamic navigation for implant placement—Choosing the method of guidance. Journal of Oral and Maxillofacial Surgery, 74(2), 269–277. 10.1016/j.joms.2015.09.022 26452429

[cre2878-bib-0006] Block, M. S. , Emery, R. W. , Cullum, D. R. , & Sheikh, A. (2017). Implant placement is more accurate using dynamic navigation. Journal of Oral and Maxillofacial Surgery, 75(7), 1377–1386. 10.1016/J.JOMS.2017.02.026 28384461

[cre2878-bib-0007] Brief, J. , Edinger, D. , Hassfeld, S. , & Eggers, G. (2005). Accuracy of image‐guided implantology. Clinical Oral Implants Research, 16(4), 495–501. 10.1111/J.1600-0501.2005.01133.X 16117776

[cre2878-bib-0008] Casap, N. , Nadel, S. , Tarazi, E. , & Weiss, E. I. (2011). Evaluation of a navigation system for dental implantation as a tool to train novice dental practitioners. Journal of Oral and Maxillofacial Surgery, 69(10), 2548–2556. 10.1016/j.joms.2011.04.026 21821328

[cre2878-bib-0009] Cassetta, M. , & Bellardini, M. (2017). How much does experience in guided implant surgery play a role in accuracy? A randomized controlled pilot study. International Journal of Oral and Maxillofacial Surgery, 46(7), 922–930. 10.1016/J.IJOM.2017.03.010 28366450

[cre2878-bib-0010] Choi, W. , Nguyen, B.‐C. , Doan, A. , Girod, S. , Gaudilliere, B. , & Gaudilliere, D. (2017). Freehand versus guided surgery. Implant Dentistry, 28(4), 500–509. 10.1097/ID.0000000000000620 28731896

[cre2878-bib-0011] Choi, Y.‐D. , Mai, H.‐N. , Mai, H. Y. , Ha, J.‐H. , Li, L.‐J. , & Lee, D.‐H. (2020). The effects of distribution of image matched fiducial markers on accuracy of computer‐guided implant surgery. Journal of Prosthodontics, 29(5), 409–414. 10.1111/jopr.13171 32237001

[cre2878-bib-0012] Čoh, M. , Jovanović‐Golubović, D. , & Milovan, B. (2004). Motor learning in sport. Facta Universitatis Physical Education and Sport, 2(1), 45–59.

[cre2878-bib-0013] Deeb, G. R. , Allen, R. K. , Hall, V. P. , Whitley, D. , Laskin, D. M. , & Bencharit, S. (2017). How accurate are implant surgical guides produced with desktop stereolithographic 3‐dimentional printers. Journal of Oral and Maxillofacial Surgery, 75(12), 2559.e1–2559.e8. 10.1016/J.JOMS.2017.08.001 28863884

[cre2878-bib-0014] Deeb, J. G. , Kanduti, D. , Skrjanc, L. , Carrico, C. K. , & Deeb, G. (2022). Comparison of accuracy and time for four implant placement techniques supporting fixed‐partial denture. Journal of Oral Implantology, 48(6), 562–572. 10.1563/aaid-joi-D-20-00415 35503961

[cre2878-bib-0015] Engkawong, S. , Mattheos, N. , Pisarnturakit, P. P. , Pimkhaokham, A. , & Subbalekha, K. (2021). Comparing patient‐reported outcomes and experiences among static, dynamic computer‐aided, and conventional freehand dental implant placement: A randomized clinical trial. Clinical Implant Dentistry and Related Research, 23(5), 660–670. 10.1111/CID.13030 34231956

[cre2878-bib-0016] Gargallo‐Albiol, J. , Barootchi, S. , Marqués‐Guasch, J. , & Wang, H.‐L. (2020). Fully guided versus half‐guided and freehand implant placement: Systematic review and meta‐analysis. The International Journal of Oral & Maxillofacial Implants, 35(6), 1159–1169. 10.11607/jomi.7942 33270056

[cre2878-bib-0017] Golob Deeb, J. , Bencharit, S. , Carrico, C. K. , Lukic, M. , Hawkins, D. , Rener‐Sitar, K. , & Deeb, G. R. (2019). Exploring training dental implant placement using computer‐guided implant navigation system for predoctoral students: A pilot study. European Journal of Dental Education, 23(4), 415–423. 10.1111/eje.12447 31141291

[cre2878-bib-0018] Hama, D. R. , & Mahmood, B. J. (2023). Comparison of accuracy between free‐hand and surgical guide implant placement among experienced and non‐experienced dental implant practitioners: An in vitro study. Journal of Periodontal & Implant Science, 53(5), 388–401. 10.5051/jpis.2204700235 37154109 PMC10627737

[cre2878-bib-0019] Harris, D. J. , Vine, S. J. , Wilson, M. R. , McGrath, J. S. , LeBel, M.‐E. , & Buckingham, G. (2018). Action observation for sensorimotor learning in surgery. British Journal of Surgery, 105(13), 1713–1720. 10.1002/bjs.10991 30259958

[cre2878-bib-0020] Kopp, S. , Warkentin, M. , Öri, F. , Ottl, P. , Kundt, G. , & Frerich, B. (2012). Section plane selection influences the results of histomorphometric studies: The example of dental implants. Biomedizinische Technik/Biomedical Engineering, 57(5), 365–370. 10.1515/BMT-2012-0015 25854664

[cre2878-bib-0021] Krakauer, J. W. , Hadjiosif, A. M. , Xu, J. , Wong, A. L. , & Haith, A. M. (2019). Motor learning. Comprehensive Physiology, 9(2), 613–663. 10.1002/cphy.c170043 30873583

[cre2878-bib-0022] Lv, H.‐X. , Rong, R. , & Sa, Y. (2023). Radiopaque fiducial markers as an aid to fabrication of an implant surgical guide for a patient with orthodontic brackets: A dental technique. The Journal of Prosthetic Dentistry, 129(6), 835–839. 10.1016/j.prosdent.2021.07.029 34556334

[cre2878-bib-0023] Neville, H. , & Bavelier, D. (2002). Human brain plasticity: Evidence from sensory deprivation and altered language experience. Progress in Brain Research, 138, 177–188. 10.1016/S0079-6123(02)38078-6 12432770

[cre2878-bib-0024] Pascual‐Leone, A. , Grafman, J. , & Hallett, M. (1994). Modulation of cortical motor output maps during development of implicit and explicit knowledge. Science, 263(5151), 1287–1289. 10.1126/science.8122113 8122113

[cre2878-bib-0025] Payer, M. , Kirmeier, R. , Jakse, N. , Pertl, C. , Wegscheider, W. , & Lorenzoni, M. (2008). Surgical factors influencing mesiodistal implant angulation. Clinical Oral Implants Research, 19(3), 265–270. 10.1111/J.1600-0501.2007.01464.X 18081867

[cre2878-bib-0026] Pellegrino, G. , Ferri, A. , Del Fabbro, M. , Prati, C. , Gandolfi, M. , & Marchetti, C. (2021). Dynamic navigation in implant dentistry: A systematic review and meta‐analysis. The International Journal of Oral & Maxillofacial Implants, 36(5), e121–e140. 10.11607/jomi.8770 34698720

[cre2878-bib-0027] Roller, C. A. , Cohen, H. S. , Kimball, K. T. , & Bloomberg, J. J. (2001). Variable practice with lenses improves visuo‐motor plasticity. Cognitive Brain Research, 12(2), 341–352. 10.1016/S0926-6410(01)00077-5 11587905

[cre2878-bib-0028] Romanos, G. E. , Delgado‐Ruiz, R. , & Sculean, A. (2019). Concepts for prevention of complications in implant therapy. Periodontology 2000, 81(1), 7–17. 10.1111/PRD.12278 31407435

[cre2878-bib-0029] Scherer, M. D. , Kattadiyil, M. T. , Parciak, E. , & Puri, S. (2014). CAD/CAM guided surgery in implant dentistry. A review of software packages and step‐by‐step protocols for planning surgical guides. The Alpha Omegan, 107(1), 32–38. 24881445

[cre2878-bib-0030] Schnutenhaus, S. , Wagner, M. , Edelmann, C. , Luthardt, R. G. , & Rudolph, H. (2021). Factors influencing the accuracy of freehand implant placement: A prospective clinical study. Dentistry Journal, 9(5), 54. 10.3390/dj9050054 34068734 PMC8151810

[cre2878-bib-0031] Seidler, R. D. (2004). Multiple motor learning experiences enhance motor adaptability. Journal of Cognitive Neuroscience, 16(1), 65–73. 10.1162/089892904322755566 15006037

[cre2878-bib-0032] Seymour, N. E. (2008). VR to OR: A review of the evidence that virtual reality simulation improves operating room performance. World Journal of Surgery, 32(2), 182–188. 10.1007/s00268-007-9307-9 18060453

[cre2878-bib-0033] Shea, C. H. , Lai, Q. , Black, C. , & Park, J.‐H. (2000). Spacing practice sessions across days benefits the learning of motor skills. Human Movement Science, 19(5), 737–760. 10.1016/S0167-9457(00)00021-X

[cre2878-bib-0034] Somogyi‐Ganss, E. , Holmes, H. I. , & Jokstad, A. (2015). Accuracy of a novel prototype dynamic computer‐assisted surgery system. Clinical Oral Implants Research, 26(8), 882–890. 10.1111/CLR.12414 24837492

[cre2878-bib-0035] Tahmaseb, A. , Wu, V. , Wismeijer, D. , Coucke, W. , & Evans, C. (2018). The accuracy of static computer‐aided implant surgery: A systematic review and meta‐analysis. Clinical Oral Implants Research, 29, 416–435. 10.1111/CLR.13346 30328191

[cre2878-bib-0036] Varga, E. , Antal, M. , Major, L. , Kiscsatári, R. , Braunitzer, G. , & Piffkó, J. (2020). Guidance means accuracy: A randomized clinical trial on freehand versus guided dental implantation. Clinical Oral Implants Research, 31(5), 417–430. 10.1111/clr.13578 31958166

[cre2878-bib-0037] Wolpert, D. M. , Diedrichsen, J. , & Flanagan, J. R. (2011). Principles of sensorimotor learning. Nature Reviews Neuroscience, 12(12), 739–751. 10.1038/nrn3112 22033537

[cre2878-bib-0038] Wulf, G. (2013). Attentional focus and motor learning: A review of 15 years. International Review of Sport and Exercise Psychology, 6(1), 77–104. 10.1080/1750984X.2012.723728

[cre2878-bib-0039] Xeroulis, G. J. , Park, J. , Moulton, C.‐A. , Reznick, R. K. , LeBlanc, V. , & Dubrowski, A. (2007). Teaching suturing and knot‐tying skills to medical students: A randomized controlled study comparing computer‐based video instruction and (concurrent and summary) expert feedback. Surgery, 141(4), 442–449. 10.1016/j.surg.2006.09.012 17383520

[cre2878-bib-0040] Yang, W.‐M. , Ho, C.‐T. , & Lo, L.‐J. (2015). Automatic superimposition of palatal fiducial markers for accurate integration of digital dental model and cone beam computed tomography. Journal of Oral and Maxillofacial Surgery, 73(8), 1616.e1–1616.e10. 10.1016/j.joms.2015.04.004 25957873

[cre2878-bib-0041] Yi, C. , Li, S. , Wen, A. , Wang, Y. , Zhao, Y. , & Zhang, Y. (2022). Digital versus radiographic accuracy evaluation of guided implant surgery: An in vitro study. BMC Oral health, 22(1), 540. 10.1186/s12903-022-02585-5 36424579 PMC9694847

